# Supplementary material for: Co‐catabolism of arginine and succinate drives symbiotic nitrogen fixation
Source: Mol Syst Biol. 2020 Jun 3;16(6):e9419. doi: 10.15252/msb.20199419 (PMC7268258; doi:10.15252/msb.20199419)
Supplement: Supplementary file 1 — Appendix [file MSB-16-e9419-s001.pdf]

Appendix

Co-catabolism of arginine and succinate drives symbiotic nitrogen fixation

Carlos Eduardo Flores-Tinoco[a], **Flavia Tschan**[a], **Tobias Fuhrer**[a], **Cèline Margot**[a], **Uwe Sauer**[a], **Matthias Christen**[a,1], **and Beat Christen**[a,1]

[a]Institute of Molecular Systems Biology, Eidgenössische Technische Hochschule (ETH) Zürich, CH-8093 Zürich, Switzerland
[1]To whom correspondence should be addressed. E-mail: matthias.christen@imsb.biol.ethz.ch; beat.christen@imsb.biol.ethz.ch

This PDF file includes:

Figures S1 to S2
Tables S1 to S8
Reaction stoichiometry of nitrogen fixation metabolism

- CATCH-N1, stochiometric model with pyruvate as the final amino-acceptor
- CATCH-N2, model with oxaloacetate (OA) as the final amino-acceptor
- CATCH-N3, stochiometric model with arginine and malate with pyruvate as the final amino-acceptor
- CATCH-N4, stochiometric model with arginine and malate with oxaloacetate as the final amino-acceptor
- Metabolism under the sole provision of arginine
- Metabolism under the assumption of an operational TCA cycle

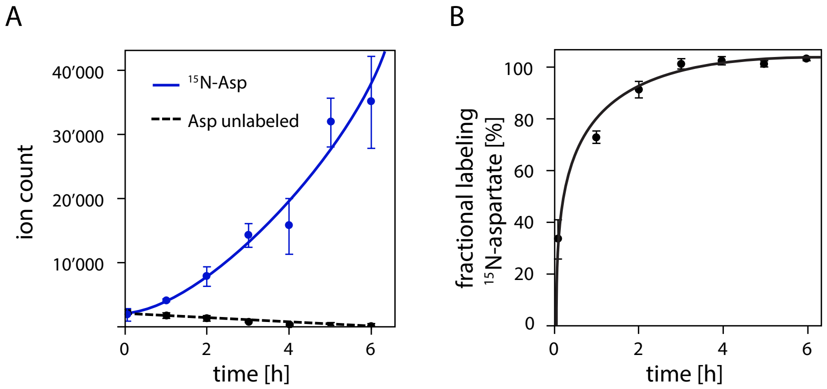

Figure S1. Production of ^{15}N-labeled aspartate in isolated *B. diazoefficiens* bacteroids. (**a**) Detection of ^{15}N-aspartate (blue) and unlabeled aspartate (dashed line) upon co-feeding of ^{15}N-arginine and succinate in isolated *B. diazoefficiens* bacteroids by MS. (**b**) Fractional labeling of ^{15}N-aspartate in the whole cell broth over the time course.

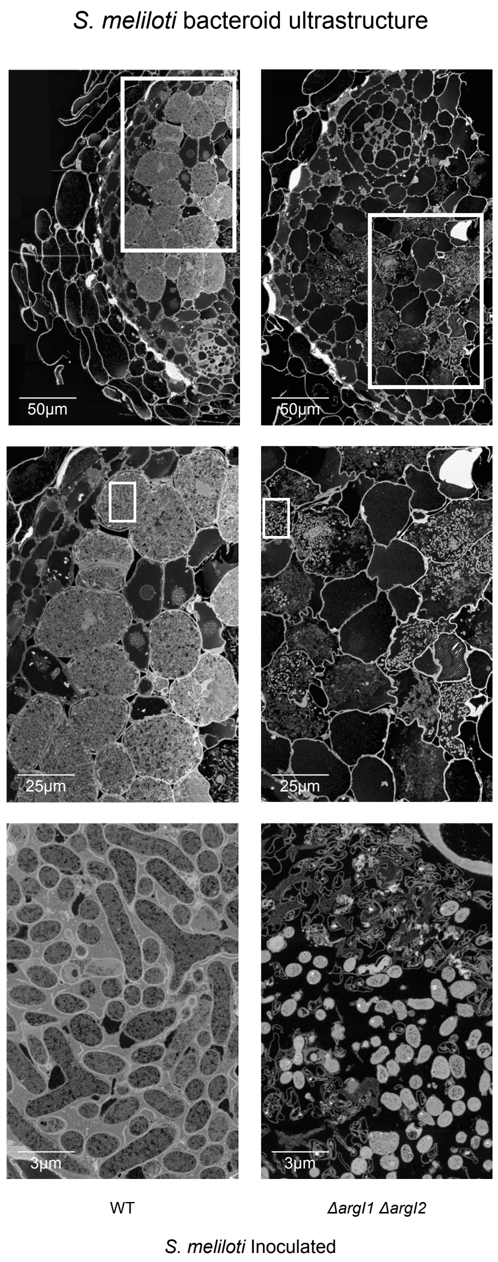

Figure S2. Arginine catabolism is fundamental for bacteroid development. Scanning electron micrographs from cross-sections of nodules bearing *S. meliloti* WT (left) or arginine catabolism double mutant Δ *argl1* Δ *argl2* (right) showing bacteroid occupancy of the nodule and ultrastructure. The white box indicates the area enlarged for the subsequent panel.

Table S1. Substrate-dependent nitrogenase activity in isolated *B. diazoefficiens* bacteroids

Substrate	Nitrogenase activity [a,c] [μmol h^{-1} g^{-1}]	Rel. Activity [b,c]	Replicates
succinate	11.28 ± 0.38	0.08 ± 0.04	17
malate	5.99 ± 0.75	-0.20 ± 0.05	7
arginine	14.6 ± 0.67	0.46 ± 0.04	8
malate arginine	9.66 ± 0.62	0.09 ± 0.04	7
succinate arginine	19.25 ± 0.69	0.91 ± 0.06	17
crude extract	20.38 ± 1.39	1.00 ± 0.07	17

[a] Mean ethylene produced
[b] Relative nitrogenase activity to mock-supplemented bacteroids
[c] Mean ± SEM

Table S2. Symbiotic N_2-fixation phenotypes of *M. truncatula* inoculated with *S. meliloti*

S. meliloti	Shoot weight[a]	Nodulation frequency[b]	Nitrogenase activity [%][c]	Replicates
WT	18.15 ± 1.42	17.95 ± 0.75	100.00 ± 7.36	109
Δ*artABCDE*	15.59 ± 1.73	28.39 ± 1.70	52.94 ± 7.27	46
Δ*satABC*	12.81 ± 1.89	25.58 ± 2.25	44.55 ± 10.99	33
Δ*argI1*	10.51 ± 0.93	27.06 ± 2.28	28.82 ± 5.21	47
Δ*argI2*	11.34 ± 1.20	26.84 ± 1.72	29.03 ± 5.16	43
Δ*argI1*Δ*argI2*	9.79 ± 1.11	36.70 ± 2.57	19.11 ± 3.15	40
Δ*ureGFE*	9.46 ± 0.62	26.43 ± 2.27	19.10 ± 4.81	30
Δ*amtB*	12.73 ± 1.25	30.97 ± 2.37	35.32 ± 5.50	31
Δ*aspC*	15.45 ± 1.65	27.75 ± 1.54	49.09 ± 9.00	40
Δ*dctAB*	7.01 ± 0.29	30.77 ± 2.14	0.00 ± 0.04	35
Δ*nifD*	6.60 ± 0.23	35.49 ± 1.67	0.00 ± 0.03	72
Non inoculated	6.31 ± 0.31	0.00 ± 0.00	n.a.[d]	42

[a] Shoot mass of *M. truncatula* plants after 8 week post inoculation. Shown is average ± SEM.

[b] Number of visible nodules per plant after 8 week post inoculation. Shown is mean ± SEM.

[c] Relative nitrogenase activity per nodule. Shown is mean ± SEM.

[d] Not applies as non inoculated controls did not form nodules.

Table S3. Substrate specificity of the transaminase AspC

Amino donor	Relative activity (%)[a]
L-Arginine	100.0 ± 4.9
Agmatine	112.6 ± 6.8
Putrescine	73.8 ± 6.8
Ornithine	64.6 ± 6.8

[a] Mean ± SEM, with $n \geqslant 4$.

Table S4. Substrate specificity of ureohydrolases

Enzyme	Locus tag [a]	V_0 [µmol min^{-1} mg^{-1}] [b]	k_{cat} [s^{-1}] [b]
EC: 3.5.3.1 [c]: Arginine ->Ornithine [d]			
ArgI1	SMc03091	0.38 ± 0.04	0.210 ± 0.023
ArgI2	SMa1711	0.01 ± 0.00	0.003 ± 0.000
SpeB2	SMc01967	0.00 ± 0.00	0.003 ± 0.000
SpeB	SMc01802	0.00 ± 0.00	0.000 ± 0.000
control		0.00 ± 0.00	0.000 ± 0.000
EC: 3.5.3.7 [c]: GOP ->AOP [d]			
SpeB2	SMc01967	1.49 ± 0.07	0.958 ± 0.044
ArgI1	SMc03091	0.01 ± 0.00	0.003 ± 0.000
ArgI2	SMa1711	0.00 ± 0.00	0.001 ± 0.000
SpeB	SMc01802	0.00 ± 0.00	−0.001 ± 0.000
control		0.00 ± 0.00	0.000 ± 0.000
EC: 3.5.3.11 [c]: Agmatine ->Putrescine [d]			
SpeB	SMc01802	3.01 ± 0.27	1.775 ± 0.159
ArgI2	SMa1711	0.04 ± 0.00	0.023 ± 0.001
SpeB2	SMc01967	0.03 ± 0.00	0.018 ± 0.003
ArgI1	SMc03091	0.00 ± 0.00	0.002 ± 0.000
control		0.00 ± 0.00	0.000 ± 0.000
EC: 3.5.3.7 [c]: 4-guanidinobutanoate ->4-aminobutanoate [d]			
SpeB2	SMc01967	1.11 ± 0.07	0.716 ± 0.048
SpeB	SMc01802	0.03 ± 0.00	0.018 ± 0.001
ArgI2	SMa1711	0.02 ± 0.00	0.009 ± 0.001
ArgI1	SMc03091	0.00 ± 0.00	0.002 ± 0.001
control		0.01 ± 0.00	0.000 ± 0.000
EC: 3.5.3.7 [c]: 4-guanidnobutanal [d]->4-aminobutanal			
SpeB2	SMc01967	2.69 ± 0.71	1.728 ± 0.459
ArgI1	SMc03091	0.01 ± 0.01	0.006 ± 0.004
SpeB	SMc01802	0.01 ± 0.00	0.003 ± 0.002
ArgI2	SMa1711	−0.01 ± 0.00	−0.005 ± 0.001
control		−0.02 ± 0.00	0.000 ± 0.000

[a] *S. meliloti* locus tag of the enzyme described
[b] Mean ± SEM, with n ⩾ 3
[c] Enzyme Commission (EC) number
[d] Ion analyzed

Table S5. Substrate specificity of dehydrogenases

Enzyme	Locus tag [a]	V_0 [µmol min^{-1} mg^{-1}] [b]	k_{cat} [s^{-1}] [b]
EC: 1.2.1.16 [b]: Succinate semialdehyde ->Succinate			
GabD6	SM_b21301	3.63 ± 0.05	5.16 ± 0.08
GabD7	SMc02689	1.09 ± 0.01	1.02 ± 0.01
GabD1	SMc02780	0.76 ± 0.00	0.66 ± 0.00
GabD8	SMa0796	0.39 ± 0.11	0.34 ± 0.10
GabD9	SM_b20424	−0.01 ± 0.00	−0.01 ± 0.00
control		0.00 ± 0.00	0.00 ± 0.00
EC: 1.2.1.54 [b]: 4-Guanidinobutanal ->4-Guanidinobutanoate			
GabD6	SM_b21301	3.37 ± 0.02	4.78 ± 0.03
GabD7	SMc02689	1.42 ± 0.12	1.33 ± 0.12
GabD1	SMc02780	0.80 ± 0.01	0.69 ± 0.01
GabD8	SMa0796	0.58 ± 0.05	0.52 ± 0.04
GabD9	SM_b20424	0.36 ± 0.00	0.32 ± 0.00
control		−0.01 ± 0.00	0.00 ± 0.00
EC: 1.2.1.19 [b]: 4-aminobutanal ->4-aminobutanoate			
GabD6	SM_b21301	3.73 ± 0.43	5.30 ± 0.60
GabD7	SMc02689	1.30 ± 0.07	1.22 ± 0.06
GabD1	SMc02780	0.88 ± 0.00	0.76 ± 0.00
GabD8	SMa0796	0.01 ± 1.44	0.00 ± 1.28
GabD9	SM_b20424	0.00 ± 2.48	0.00 ± 2.19
control		0.00 ± 0.00	0.00 ± 0.00

[a] *S. meliloti* locus tag of the enzyme described
[b] Mean ± SEM, with n ⩾ 3
[c] Enzyme Commission (EC) number of the reaction analyzed

Table S6. Substrate specificity of characterized transaminases

Enzyme	Locus tag [a]	V_0 [µmol min^{-1} mg^{-1}] [b]	k_{cat} [s^{-1}] [b]
EC: 2.6.1.- [c]: Pyruvate ->Alanine [d]			
AspC	SMc02262	2.705 ± 0.181	1.941 ± 0.130
DatA	SMc02251	0.380 ± 0.068	0.274 ± 0.049
GabT2	SM_b20423	0.207 ± 0.010	0.172 ± 0.008
AatB	SMc04386	0.213 ± 0.013	0.161 ± 0.010
ArgD	SMc02138	0.120 ± 0.007	0.088 ± 0.005
GabT3	SMc04388	0.102 ± 0.007	0.082 ± 0.006
control		–0.008 ± 0.001	0.000 ± 0.000
EC: 2.6.1.84 [c]: Arginine ->GOP [d]			
AspC	SMc02262	0.287 ± 0.014	0.206 ± 0.010
GabT2	SM_b20423	0.005 ± 0.000	0.004 ± 0.000
AatB	SMc04386	0.002 ± 0.000	0.002 ± 0.000
ArgD	SMc02138	0.001 ± 0.000	0.001 ± 0.000
DatA	SMc02251	0.001 ± 0.000	0.000 ± 0.000
control		0.000 ± 0.000	0.000 ± 0.000
EC: 2.6.1.- [c]: Ornithine ->AOP [d]			
AspC	SMc02262	0.186 ± 0.010	0.133 ± 0.007
DatA	SMc02251	0.092 ± 0.009	0.066 ± 0.007
AatB	SMc04386	0.070 ± 0.001	0.052 ± 0.000
ArgD	SMc02138	0.013 ± 0.000	0.009 ± 0.000
GabT2	SM_b20423	0.005 ± 0.000	0.004 ± 0.000
control		0.000 ± 0.000	0.000 ± 0.000
EC: 2.6.1.- [c]: Agmatine ->4-Guanidinobutanal [d]			
AspC	SMc02262	0.323 ± 0.019	0.232 ± 0.014
GabT2	SM_b20423	0.005 ± 0.000	0.004 ± 0.000
AatB	SMc04386	0.003 ± 0.000	0.002 ± 0.000
ArgD	SMc02138	0.001 ± 0.000	0.001 ± 0.000
DatA	SMc02251	0.001 ± 0.000	0.001 ± 0.000
control		0.000 ± 0.000	0.000 ± 0.000
EC: 2.6.1.113 [c]: Putrescine ->4-aminobutanal [d]			
AspC	SMc02262	0.212 ± 0.020	0.152 ± 0.014
AatB	SMc04386	0.033 ± 0.001	0.025 ± 0.001
DatA	SMc02251	0.034 ± 0.001	0.025 ± 0.001
GabT2	SM_b20423	0.006 ± 0.000	0.005 ± 0.000
ArgD	SMc02138	0.007 ± 0.000	0.005 ± 0.000
control		0.000 ± 0.000	0.000 ± 0.000
EC: 2.6.1.96 [c]: GABA ->SSA [d]			
GabT2	SM_b20423	0.099 ± 0.005	0.082 ± 0.004
GabT3	SMc04388	0.060 ± 0.003	0.049 ± 0.002
ArgD	SMc02138	0.010 ± 0.001	0.008 ± 0.001
control		0.001 ± 0.000	0.000 ± 0.000

[a] *S. meliloti* locus tag of the enzyme described
[b] Mean ± SEM, with n $\geqslant$ 3
[c] Enzyme Comission (EC) number
[d] Ion analyzed

Table S7. Substrate specificity of decarboxylases

Enzyme	Locus tag [a]	V_0 [µmol min^{-1} mg^{-1}] [b]	k_{cat} [s^{-1}] [b]
EC: 4.1.1.17 [c]: Ornithine ->Putrescine [d]			
OdcA	SMc02983	3.33 ± 0.28	2.32 ± 0.20
IlvB1	SMc02263	0.00 ± 0.00	0.00 ± 0.00
OdcB	SMa0682	0.00 ± 0.00	0.00 ± 0.00
control		−0.01 ± 0.00	0.00 ± 0.00
EC: 4.1.1.19 [c]: Arginine ->Agmatine [d]			
OdcA	SMc02983	0.23 ± 0.02	0.16 ± 0.02
OdcB	SMa0682	0.00 ± 0.01	0.00 ± 0.02
IlvB1	SMc02263	−0.04 ± 0.01	−0.04 ± 0.01
control		−0.01 ± 0.00	0.00 ± 0.00
EC: 4.1.1.75 [c]: GOP ->4-Guanidinobutanal [d]			
IlvB1	SMc02263	0.33 ± 0.00	0.31 ± 0.00
OdcA	SMc02983	−0.02 ± 0.00	−0.01 ± 0.00
OdcB	SMa0682	−0.02 ± 0.00	−0.02 ± 0.00
control		−0.04 ± 0.00	0.00 ± 0.00
EC: 4.1.1.75 [c]: AOP ->Pyrroline [de]			
IlvB1	SMc02263	2.31 ± 0.36	2.16 ± 0.33
OdcB	SMa0682	0.12 ± 0.03	0.17 ± 0.04
OdcA	SMc02983	0.01 ± 0.00	0.00 ± 0.00
control		0.01 ± 0.00	0.00 ± 0.00

[a] *S. meliloti* locus tag of the enzyme described
[b] Mean ± SEM, with n $\geqslant$ 3
[c] Enzyme Comission (EC) number
[d] Ion analyzed
[e] Pyrroline is the spontaneous cyclic form of 4-aminobutanal (ABL)

Table S8. Relative ion abundance during synthetic reconstitution of arginine catabolism

Time [min]	ARG	ORN	GOP	AOP	PUT	GBL	GABA	SUCC	ALA
0	1.00 ± 0.00	0.00 ± 0.00	0.00 ± 0.00	0.00 ± 0.00	0.00 ± 0.00	0.00 ± 0.00	0.00 ± 0.00	0.00 ± 0.00	0.00 ± 0.00
1	0.84 ± 0.01	0.57 ± 0.05	0.61 ± 0.06	0.03 ± 0.02	0.09 ± 0.04	0.62 ± 0.07	0.08 ± 0.05	0.09 ± 0.07	0.00 ± 0.02
2	0.69 ± 0.02	0.82 ± 0.03	0.57 ± 0.02	0.00 ± 0.01	0.05 ± 0.03	0.42 ± 0.06	0.15 ± 0.05	0.09 ± 0.08	0.02 ± 0.03
5	0.29 ± 0.01	0.90 ± 0.02	0.83 ± 0.06	0.09 ± 0.01	0.12 ± 0.02	0.60 ± 0.01	0.08 ± 0.06	0.00 ± 0.07	0.09 ± 0.01
10	0.13 ± 0.01	1.00 ± 0.01	1.00 ± 0.10	0.14 ± 0.03	0.20 ± 0.03	1.00 ± 0.22	0.24 ± 0.07	0.12 ± 0.05	0.15 ± 0.02
30	0.09 ± 0.03	0.92 ± 0.05	0.21 ± 0.02	0.35 ± 0.02	0.87 ± 0.10	0.37 ± 0.08	0.69 ± 0.09	0.00 ± 0.08	0.24 ± 0.02
60	0.05 ± 0.01	0.77 ± 0.06	0.00 ± 0.03	0.49 ± 0.00	0.92 ± 0.09	0.00 ± 0.01	0.84 ± 0.08	0.36 ± 0.12	0.27 ± 0.02
90	0.03 ± 0.01	0.63 ± 0.06	0.00 ± 0.02	0.61 ± 0.01	0.86 ± 0.08	0.05 ± 0.02	0.69 ± 0.03	0.25 ± 0.04	0.40 ± 0.02
120	0.05 ± 0.02	0.47 ± 0.04	0.00 ± 0.11	0.74 ± 0.02	0.97 ± 0.14	0.06 ± 0.09	0.96 ± 0.15	0.65 ± 0.12	0.57 ± 0.05
150	0.08 ± 0.02	0.75 ± 0.04	0.00 ± 0.04	0.71 ± 0.01	1.00 ± 0.16	0.00 ± 0.06	1.00 ± 0.07	0.45 ± 0.03	0.69 ± 0.02
180	0.06 ± 0.02	0.69 ± 0.07	0.00 ± 0.02	0.82 ± 0.01	0.93 ± 0.11	0.00 ± 0.05	0.77 ± 0.05	0.61 ± 0.01	0.79 ± 0.03
240	0.00 ± 0.01	0.55 ± 0.04	0.00 ± 0.03	1.00 ± 0.01	0.70 ± 0.10	0.01 ± 0.03	0.54 ± 0.04	1.00 ± 0.16	1.00 ± 0.04

[a] The names of the metabolites are indicated above their chemical structures according to the following abbreviations: arginine (ARG), ornithine (ORN), 5-guanidino-2-oxo-pentanoate (GOP), 5-amino-oxopentanoate (AOP), putrescine (PUT) , 4-guanidino-butyraldehyde (GBL), 4-aminobutanoate (GABA), succinate (SUCC) and alanine (ALA)

[b] Mean ± SEM, with n $\geqslant$ 3

Reaction stoichiometry of nitrogen fixation metabolism upon co-feeding of arginine and succinate. . Reaction equations are step-wise deduced from the conversion of arginine into gamma-amino butanoic acid (GABA), GABA to Succinate and conversion to Pyruvate. Furthermore, reaction equations are deduced from electron bifurcation of NADH by the FixABCX complex, nitrogen fixation by nitrogenase complex and oxidative phosphorylation by the FbcBCF and the high-affine terminal cbb3 oxidases FixNOPQ1-3.

CATCH-N1, stochiometric model with pyruvate as the final amino-acceptor

Arginine to GABA:

$$\mathrm{Arg} + \mathrm{NAD} + \mathrm{Pyr} + 3\,\mathrm{H_2O} \longrightarrow \mathrm{GABA} + \mathrm{Ala} + \mathrm{NADH_2} + 2\,\mathrm{NH_3} + 2\,\mathrm{CO_2}, \qquad [1]$$

1 ATP generated and 3 H^+ consumed per arginine through the carbamoyl pathway [2]

GABA to succinate:

$$\mathrm{GABA} + \mathrm{Pyr} + \mathrm{NAD} + \mathrm{H_2O} \longrightarrow \mathrm{Suc} + \mathrm{Ala} + \mathrm{NADH_2} \qquad [3]$$

Arginine to succinate, 1 + 3:

$$\mathrm{Arg} + 2\,\mathrm{NAD} + 2\,\mathrm{Pyr} + 4\,\mathrm{H_2O} \longrightarrow \mathrm{Suc} + 2\,\mathrm{Ala} + 2\,\mathrm{NADH_2} + 2\,\mathrm{NH_3} + 2\,\mathrm{CO_2} \qquad [4]$$

Succinate to pyruvate:

$$2\,\mathrm{Suc} + 2\,\mathrm{Q} + 2\,\mathrm{NAD} + 2\,\mathrm{H_2O} \longrightarrow 2\,\mathrm{Pyr} + 2\,\mathrm{NADH_2} + 2\,\mathrm{QH_2} + 2\,\mathrm{CO_2} \qquad [5]$$

Co-catabolism of arginine and succinate to 2 alanine: 4 + 5:

$$\mathrm{Arg} + \mathrm{Suc} + 4\,\mathrm{NAD} + 2\,\mathrm{Q} + 6\,\mathrm{H_2O} \longrightarrow 2\,\mathrm{Ala} + 4\,\mathrm{NADH_2} + 2\,\mathrm{QH_2} + 2\,\mathrm{NH_3} + 4\,\mathrm{CO_2} \qquad [6]$$

Electron bifurcation of NADH by the FixABCX complex:

$$8\,\mathrm{NADH_2} + 8\,\mathrm{Fd_{ox}} + 4\,\mathrm{Q} \longrightarrow 8\,\mathrm{NAD} + 8\,\mathrm{Fd_{red}} + 4\,\mathrm{QH_2} \qquad [7]$$

Nitrogenase reaction by NifDK (as biochemical equation not balanced for H^+):

$$\mathrm{N_2} + 8\,\mathrm{Fd_{red}} \longrightarrow 2\,\mathrm{NH_3(aq)} + \mathrm{H_2} + 8\,\mathrm{Fd_{ox}}, \qquad \text{requires } 8\,\mathrm{H^+}, 16\text{ ATP} \qquad [8]$$

Oxidative phosphorylation by FbcBCF and high-affine terminal oxidase FixNOPQ and:

$$8\,\mathrm{QH_2} + 4\,\mathrm{O_2} \longrightarrow 8\,\mathrm{Q} + 8\,\mathrm{H_2O} \qquad (8 \cdot 6\,\mathrm{H^+} = 48\,\mathrm{H^+}\text{ exported}) \qquad [9]$$

Overall biochemical reaction, not yet balanced for charge and H^+:

$$2\,\mathrm{Arg} + 2\,\mathrm{Suc} + \mathrm{N_2} + 4\,\mathrm{O_2} + 4\,\mathrm{H_2O} \longrightarrow 4\,\mathrm{Ala} + 6\,\mathrm{NH_3} + 8\,\mathrm{CO_2} + \mathrm{H_2} \qquad \text{biochem. eq.} \qquad [10]$$

Balancing the reaction stoichiometry for charge and H^+:

$$2\,\mathrm{C_6H_{15}N_4O_2}^{+} + 2\,\mathrm{C_4H_4O_4}^{2-} + \mathrm{N_2} + 4\,\mathrm{O_2} + 4\,\mathrm{H_2O} \longrightarrow 4\,\mathrm{C_3H_7NO_2} + 6\,\mathrm{NH_4}^{+} + 8\,\mathrm{CO_2} + \mathrm{H_2} \qquad \text{chemical eq.} \qquad [11]$$

$$20\,\mathrm{C} = 20\,\mathrm{C} \qquad \text{C balanced} \qquad [12]$$

$$10\,\mathrm{N} = 10\,\mathrm{N} \qquad \text{N balanced} \qquad [13]$$

$$24\,\mathrm{O} = 24\,\mathrm{O} \qquad \text{O balanced} \qquad [14]$$

$$46\,\mathrm{H} \neq 54\,\mathrm{H} \qquad 8\,\mathrm{H^+}\text{missing} \qquad [15]$$

$$-2 \neq +6 \qquad +8\text{ missing} \qquad [16]$$

Insertion of 8 H^+ to the left of 11 results in the following charge and H^+ corrected equation:

$$2\,\mathrm{C_6H_{15}N_4O_2}^{+} + 2\,\mathrm{C_4H_4O_4}^{2-} + \mathrm{N_2} + 4\,\mathrm{O_2} + 4\,\mathrm{H_2O} + 8\,\mathrm{H^+} \longrightarrow 4\,\mathrm{C_3H_7NO_2} + 6\,\mathrm{NH_4}^{+} + 8\,\mathrm{CO_2} + \mathrm{H_2} \qquad \text{chemical eq.} \qquad [17]$$

Proton translocation:

$$48\,\mathrm{H^+}_{\mathrm{in}} \longrightarrow 48\,\mathrm{H^+}_{\mathrm{out}} \qquad \mathrm{O_2}\text{ dependent proton translocation from 9} \qquad [18]$$

$$48\,\mathrm{H^+}_{\mathrm{out}} \longrightarrow 48\,\mathrm{H^+}_{\mathrm{in}} \qquad \text{protons available from 9} \qquad [19]$$

$$8\,\mathrm{H^+}_{\mathrm{out}} \longrightarrow 8\,\mathrm{H^+}_{\mathrm{in}} \qquad \text{influx of } \mathrm{H^+} \text{ derived from 17} \qquad [20]$$

$$56\,\mathrm{H^+}_{\mathrm{out}} \longrightarrow 56\,\mathrm{H^+}_{\mathrm{in}} \qquad \text{19 + 20 } \textbf{Sum of proton influx} \qquad [21]$$

Stochiometry of the proton motive force (PMF) dependent ATP synthesis:

$$\frac{[\mathrm{H^+}]}{[\mathrm{ATP}]} = \frac{10}{3} \qquad , \frac{56}{3.33} = 16.8\text{, ATP synthesized} \qquad [22]$$

Stoichiometry of Enzyme-coupled ATP synthesis:

2 ATP/ 2 arginine generated by the action of deiminase, transcarbamoylase and carbamate kinase, 1 [23]

Sum of oxygen-dependent, oxygen-independent and Enzyme-coupled ATP synthesis - :

14.40 ATP Oxygen-dependent [24]

2.40 ATP Oxygen-independent [25]

2 ATP Enzyme-coupled [26]

18.8 ATP Sum [27]

-16 ATP Nitrogenase reaction [28]

2.8 ATP Net gain [29]

[30]

Overall reaction of N_2 fixation by bacteroid:

$$2\,\mathrm{Arg} + 2\,\mathrm{Suc} + \mathrm{N_2} + 4\,\mathrm{O_2} + n\,\mathrm{ADP} + n\,\mathrm{Pi} + \text{(4-n)}\,\mathrm{H_2O} + 8\,\mathrm{H^+} \longrightarrow 4\,\mathrm{Ala} + 6\,\mathrm{NH_4}^+ + 8\,\mathrm{CO_2} + \mathrm{H_2} + \mathrm{nATP} \quad [31]$$

with n= 2.80 [32]

Reaction stoichiometry of nitrogen fixation metabolism upon co-feeding of arginine and succinate. . Reaction equations are step-wise deduced from the conversion of arginine into gamma-amino butanoic acid (GABA), GABA to Succinate and conversion to oxaloacetate. Furthermore, reaction equations are deduced from electron bifurcation of NADH by the FixABCX complex, nitrogen fixation by nitrogenase complex and oxidative phosphorylation by the FbcBCF and the high-affine terminal cbb3 oxidases FixNOPQ1-3.

CATCH-N2, model with oxaloacetate (OA) as the final amino-acceptor

Arginine to GABA with oxaloacetate as amino-acceptor:

$$\mathrm{Arg} + \mathrm{NAD} + \mathrm{OA} + 3\,\mathrm{H_2O} \longrightarrow \mathrm{GABA} + \mathrm{Asp} + \mathrm{NADH_2} + 2\,\mathrm{NH_3} + \mathrm{CO_2}, \quad [33]$$

1 ATP generated and 3 H^+ consumed per arginine through the carbamoyl pathway [34]

GABA to succinate:

$$\mathrm{GABA} + \mathrm{OA} + \mathrm{NAD} + \mathrm{H_2O} \longrightarrow \mathrm{Suc} + \mathrm{Asp} + \mathrm{NADH_2} \quad [35]$$

Arginine to succinate, 33 + 35:

$$\mathrm{Arg} + 2\,\mathrm{NAD} + 2\,\mathrm{OA} + 4\,\mathrm{H_2O} \longrightarrow \mathrm{Suc} + 2\,\mathrm{Asp} + 2\,\mathrm{NADH_2} + 2\,\mathrm{NH_3} + 2\,\mathrm{CO_2} \quad [36]$$

Succinate to oxaloacetate:

$$2\,\mathrm{Suc} + 2\,\mathrm{Q} + 2\,\mathrm{NAD} + 2\,\mathrm{H_2O} \longrightarrow 2\,\mathrm{OA} + 2\,\mathrm{NADH_2} + 2\,\mathrm{QH_2} \quad [37]$$

Co-catabolism of arginine and succinate to 2 asparatate: 36 + 37:

$$\mathrm{Arg} + \mathrm{Suc} + 4\,\mathrm{NAD} + 2\,\mathrm{Q} + 6\,\mathrm{H_2O} \longrightarrow 2\,\mathrm{Asp} + 4\,\mathrm{NADH_2} + 2\,\mathrm{QH_2} + 2\,\mathrm{NH_3} + 2\,\mathrm{CO_2} \quad [38]$$

Electron bifurcation of NADH by the FixABCX complex:

$$8\,\mathrm{NADH_2} + 8\,\mathrm{Fd_{ox}} + 4\,\mathrm{Q} \longrightarrow 8\,\mathrm{NAD} + 8\,\mathrm{Fd_{red}} + 4\,\mathrm{QH_2} \quad [39]$$

Nitrogenase reaction by NifDK (as biochemical equation not balanced for H^+):

$$\mathrm{N_2} + 8\,\mathrm{Fd_{red}} \longrightarrow 2\,\mathrm{NH_3(aq)} + \mathrm{H_2} + 8\,\mathrm{Fd_{ox}}, \qquad \text{requires } 8\,\mathrm{H^+},\ 16\ \mathrm{ATP} \quad [40]$$

Oxidative phosphorylation by FbcBCF and high-affine terminal oxidase FixNOPQ:

$$8\,\mathrm{QH_2} + 4\,\mathrm{O_2} \longrightarrow 8\,\mathrm{Q} + 8\,\mathrm{H_2O} \qquad (8 \cdot 6\,\mathrm{H^+} = 48\,\mathrm{H^+}\ \text{exported}) \quad [41]$$

Overall biochemical reaction, not yet balanced for charge and H^+:

$$2\,\mathrm{Arg} + 2\,\mathrm{Suc} + \mathrm{N_2} + 4\,\mathrm{O_2} + 4\,\mathrm{H_2O} \longrightarrow 4\,\mathrm{Asp} + 6\,\mathrm{NH_3} + 4\,\mathrm{CO_2} + \mathrm{H_2} \qquad \text{biochem. eq.} \quad [42]$$

Balancing the reaction stoichiometry for charge and H^+:

$$2\,\mathrm{C_6H_{15}N_4O_2}^{+} + 2\,\mathrm{C_4H_4O_4}^{2-} + \mathrm{N_2} + 4\,\mathrm{O_2} + 4\,\mathrm{H_2O} \longrightarrow 4\,\mathrm{C_4H_6NO_4}^{-} + 6\,\mathrm{NH_4}^{+} + 4\,\mathrm{CO_2} + \mathrm{H_2} \qquad \text{chemical eq.} \quad [43]$$
$$20\,\mathrm{C} = 20\,\mathrm{C} \qquad \text{C balanced} \quad [44]$$
$$10\,\mathrm{N} = 10\,\mathrm{N} \qquad \text{N balanced} \quad [45]$$
$$24\,\mathrm{O} = 24\,\mathrm{O} \qquad \text{O balanced} \quad [46]$$
$$46\,\mathrm{H} \neq 50\,\mathrm{H} \qquad 4\,\mathrm{H^+}\text{missing} \quad [47]$$
$$-2 \neq +2 \qquad +4\ \text{missing} \quad [48]$$

Insertion of 4 H^+ to the left of 43 results in the following charge and H^+ corrected equation:

$$2\,\mathrm{C_6H_{15}N_4O_2}^{+} + 2\,\mathrm{C_4H_4O_4}^{2-} + \mathrm{N_2} + 4\,\mathrm{O_2} + 4\,\mathrm{H_2O} + 4\,\mathrm{H^+} \longrightarrow 4\,\mathrm{C_4H_6NO_4}^{-} + 6\,\mathrm{NH_4}^{+} + 4\,\mathrm{CO_2} + \mathrm{H_2} \qquad \text{chemical eq.} \quad [49]$$

Proton translocation:

$$48\,\mathrm{H^+}_{\mathrm{in}} \longrightarrow 48\,\mathrm{H^+}_{\mathrm{out}} \qquad \mathrm{O_2}\ \text{dependent proton translocation from 41} \quad [50]$$
$$48\,\mathrm{H^+}_{\mathrm{out}} \longrightarrow 48\,\mathrm{H^+}_{\mathrm{in}} \qquad \text{protons available from 41} \quad [51]$$
$$4\,\mathrm{H^+}_{\mathrm{out}} \longrightarrow 4\,\mathrm{H^+}_{\mathrm{in}} \qquad \text{influx of } \mathrm{H^+} \text{ derived from 49} \quad [52]$$
$$52\,\mathrm{H^+}_{\mathrm{out}} \longrightarrow 52\,\mathrm{H^+}_{\mathrm{in}} \qquad \text{51 + 52 } \textbf{Sum of proton influx} \quad [53]$$

Stochiometry of the proton motive force (PMF) dependent ATP synthesis:

$$\frac{[\mathrm{H^+}]}{[\mathrm{ATP}]} = \frac{10}{3} \qquad , \frac{52}{3.33} = 15.6,\ \text{ATP synthesized} \quad [54]$$

Stoichiometry of Enzyme-coupled ATP synthesis:

2 ATP/ 2 Arginine generated by deiminase, transcarbamoylase and carbamate kinase, 33 [55]

Sum of oxygen-dependent, oxygen-independent and Enzyme-coupled ATP synthesis - :

14.4 ATP Oxygen-dependent [56]
1.2 ATP Oxygen-independent [57]
2 ATP Enzyme-coupled [58]
17.60 ATP Sum [59]
-16 ATP Nitrogenase reaction [60]
1.6 ATP Net gain [61]
[62]

Overall reaction of N_2 fixation by bacteroid:

$$2\,\mathrm{Arg} + 2\,\mathrm{Suc} + \mathrm{N_2} + 4\,\mathrm{O_2} + n\,\mathrm{ADP} + n\,\mathrm{Pi} + \text{(4-n)}\,\mathrm{H_2O} + 8\,\mathrm{H^+} \longrightarrow 4\,\mathrm{Asp} + 6\,\mathrm{NH_4}^+ + 4\,\mathrm{CO_2} + \mathrm{H_2} + \mathrm{nATP} \quad [63]$$

with n= 1.6 [64]

Combined model with pyruvate and oxaloacetate as the final amino-acceptor:

$$2\,\mathrm{Arg} + 2\,\mathrm{Suc} + \mathrm{N_2} + 4\,\mathrm{O_2} + \mathrm{nADP} + \mathrm{nPi} + \text{(4-n)}\mathrm{H_2O} + \text{(8-m)}\mathrm{H^+} \longrightarrow \mathrm{mAsp} + \text{(4-m)}\mathrm{Ala} + 6\,\mathrm{NH_4} + \text{(8-m)}\mathrm{CO_2} + \mathrm{H_2} + \mathrm{nATP} \quad [65]$$

with n=(14-m)/3.33 and m=0,1,2,3,4 [66]

Reaction stoichiometry of nitrogen fixation metabolism upon co-feeding of arginine and malate. . Reaction equations are step-wise deduced from the conversion of arginine into gamma-amino butanoic acid (GABA), GABA to Succinate and conversion to Pyruvate. Furthermore, reaction equations are deduced from electron bifurcation of NADH by the FixABCX complex, nitrogen fixation by nitrogenase complex and oxidative phosphorylation by the FbcBCF and the high-affine terminal cbb3 oxidases FixNOPQ1-3.

CATCH-N3, stochiometric model with arginine and malate with pyruvate as the final amino-acceptor
Arginine to GABA:

$$\mathrm{Arg + NAD + Pyr + 3\,H_2O \longrightarrow GABA + Ala + NADH_2 + 2\,NH_3 + 2\,CO_2,} \quad [67]$$

1 ATP generated and 3 H^+ consumed per arginine through the carbamoyl pathway [68]

GABA to succinate:

$$\mathrm{GABA + Pyr + NAD + H_2O \longrightarrow Suc + Ala + NADH_2} \quad [69]$$

Arginine to succinate, 67 + 69:

$$\mathrm{Arg + 2\,NAD + 2\,Pyr + 4\,H_2O \longrightarrow Suc + 2\,Ala + 2\,NADH_2 + 2\,NH_3 + 2\,CO_2} \quad [70]$$

Succinate to Malate:

$$\mathrm{Suc + Q + H_2O \longrightarrow Mal + QH_2} \quad [71]$$

Malate to Pyruvate:

$$\mathrm{2\,Mal + 2\,NAD \longrightarrow 2\,Pyr + 2\,NADH_2 + 2\,CO_2} \quad [72]$$

Co-catabolism of arginine and malate to 2 alanine: 70 + 71 + 72:

$$\mathrm{Arg + Mal + 4\,NAD + Q + 5\,H_2O \longrightarrow 2\,Ala + 4\,NADH_2 + QH_2 + 2\,NH_3 + 4\,CO_2} \quad [73]$$

Electron bifurcation of NADH by the FixABCX complex:

$$\mathrm{8\,NADH_2 + 8\,Fd_{ox} + 4\,Q \longrightarrow 8\,NAD + 8\,Fd_{red} + 4\,QH_2} \quad [74]$$

Nitrogenase reaction by NifDK (as biochemical equation not balanced for H^+):

$$\mathrm{N_2 + 8\,Fd_{red} \longrightarrow 2\,NH_3(aq) + H_2 + 8\,Fd_{ox},} \quad \text{requires 8 H}^+\text{, 16 ATP} \quad [75]$$

Oxidative phosphorylation by FbcBCF and high-affine terminal oxidase FixNOPQ:

$$\mathrm{6\,QH_2 + 3\,O_2 \longrightarrow 6\,Q + 6\,H_2O} \quad (6\cdot 6\,\mathrm{H^+} = 36\,\mathrm{H^+}\text{ exported}) \quad [76]$$

Overall biochemical reaction, not yet balanced for charge and H^+:

$$\mathrm{2\,Arg + 2\,Mal + N_2 + 3\,O_2 + 4\,H_2O \longrightarrow 4\,Ala + 6\,NH_3 + 8\,CO_2 + H_2} \quad \text{biochem. eq.} \quad [77]$$

Balancing the reaction stoichiometry for charge and H^+:

$$\mathrm{2\,C_6H_{15}N_4O_2{}^{+} + 2\,C_4H_4O_5{}^{2-} + N_2 + 3\,O_2 + 4\,H_2O \longrightarrow 4\,C_3H_7NO_2 + 6\,NH_4{}^{+} + 8\,CO_2 + H_2} \quad \text{chemical eq.} \quad [78]$$
$$\mathrm{20\,C = 20\,C} \quad \text{C balanced} \quad [79]$$
$$\mathrm{10\,N = 10\,N} \quad \text{N balanced} \quad [80]$$
$$\mathrm{24\,O = 24\,O} \quad \text{O balanced} \quad [81]$$
$$\mathrm{46\,H \neq 54\,H} \quad \text{8 H}^+\text{missing} \quad [82]$$
$$-2 \neq +6 \quad \text{+ 8 missing} \quad [83]$$

Insertion of 8 H^+ to the left of 78 results in the following charge and H^+ corrected equation:

$$\mathrm{2\,C_6H_{15}N_4O_2{}^{+} + 2\,C_4H_4O_5{}^{2-} + N_2 + 3\,O_2 + 4\,H_2O + 8\,H^{+} \longrightarrow 4\,C_3H_7NO_2 + 6\,NH_4{}^{+} + 8\,CO_2 + H_2} \quad \text{chemical eq.} \quad [84]$$

Proton translocation:

$$\mathrm{36\,H^{+}{}_{in} \longrightarrow 36\,H^{+}{}_{out}} \quad \text{O}_2\text{ dependent proton translocation from 76} \quad [85]$$
$$\mathrm{36\,H^{+}{}_{out} \longrightarrow 36\,H^{+}{}_{in}} \quad \text{protons available from 76} \quad [86]$$
$$\mathrm{8\,H^{+}{}_{out} \longrightarrow 8\,H^{+}{}_{in}} \quad \text{influx of H}^+\text{ derived from 84} \quad [87]$$
$$\mathrm{44\,H^{+}{}_{out} \longrightarrow 44\,H^{+}{}_{in}} \quad \text{86 + 87 } \textbf{Sum of proton influx} \quad [88]$$

Stochiometry of the proton motive force (PMF) dependent ATP synthesis:

$$\frac{[\mathrm{H^+}]}{[\mathrm{ATP}]} = \frac{10}{3} \quad , \frac{44}{3.33} = 13.2\text{, ATP synthesized} \quad [89]$$

Stoichiometry of Enzyme-coupled ATP synthesis:

2 ATP/ 2 Arginine generated by the action of deiminase, transcarbamoylase and carbamate kinase, 67 [90]

Sum of oxygen-dependent, oxygen-independent and Enzyme-coupled ATP synthesis - :

10.80 ATP Oxygen-dependent [91]

2.40 ATP Oxygen-independent [92]

2 ATP Enzyme-coupled [93]

15.2 ATP Sum [94]

-16 ATP Nitrogenase reaction [95]

-0.8 ATPNet loss [96]

[97]

Overall reaction of N_2 fixation by bacteroid:

$$2\,\mathrm{Arg} + 2\,\mathrm{Mal} + \mathrm{N_2} + 3\,\mathrm{O_2} + n\,\mathrm{ADP} + n\,\mathrm{Pi} + \text{(4-n)}\,\mathrm{H_2O} + 8\,\mathrm{H^+} \longrightarrow 4\,\mathrm{Ala} + 6\,\mathrm{NH_4}^+ + 8\,\mathrm{CO_2} + \mathrm{H_2} + \mathrm{nATP} \quad [98]$$

with n= -0.8 [99]

Reaction stoichiometry of nitrogen fixation metabolism upon co-feeding of arginine and malate. . Reaction equations are step-wise deduced from the conversion of arginine into gamma-amino butanoic acid (GABA), GABA to Succinate and conversion to oxaloacetate. Furthermore, reaction equations are deduced from electron bifurcation of NADH by the FixABCX complex, nitrogen fixation by nitrogenase complex and oxidative phosphorylation by the FbcBCF and the high-affine terminal cbb3 oxidases FixNOPQ1-3.

CATCH-N4, stochiometric model with arginine and malate with oxaloacetate as the final amino-acceptor

Arginine to GABA with oxaloacetate as amino-acceptor:

$$\mathrm{Arg + NAD + OA + 3\,H_2O \longrightarrow GABA + Asp + NADH_2 + 2\,NH_3 + CO_2}, \quad [100]$$

1 ATP generated and 3 H^+ consumed per Arginine through the carbamoyl pathway [101]

GABA to succinate:

$$\mathrm{GABA + OA + NAD + H_2O \longrightarrow Suc + Asp + NADH_2} \quad [102]$$

Arginine to succinate, 100 + 102:

$$\mathrm{Arg + 2\,NAD + 2\,OA + 4\,H_2O \longrightarrow Suc + 2\,Asp + 2\,NADH_2 + 2\,NH_3 + 2\,CO_2} \quad [103]$$

Succinate to malate:

$$\mathrm{Suc + Q + H_2O \longrightarrow Mal + QH_2} \quad [104]$$

Malate to oxaloacetate:

$$\mathrm{2\,Mal + 2\,NAD \longrightarrow 2\,OA + 2\,NADH_2} \quad [105]$$

Co-catabolism of arginine and malate to 2 aspartate: 103 + 104 + 105:

$$\mathrm{Arg + Mal + 4\,NAD + Q + 5\,H_2O \longrightarrow 2\,Asp + 2\,NH_3 + 2\,CO_2 + 4\,NADH_2 + QH_2} \quad [106]$$

multiply 106 by 2 [107]

$$\mathrm{2\,Arg + 2\,Mal + 8\,NAD + 2\,Q + 10\,H_2O \longrightarrow 4\,Asp + 4\,NH_3 + 4\,CO_2 + 8\,NADH_2 + 2\,QH_2} \quad [108]$$

Electron bifurcation of NADH by the FixABCX complex:

$$\mathrm{8\,NADH_2 + 8\,Fd_{ox} + 4\,Q \longrightarrow 8\,NAD + 8\,Fd_{red} + 4\,QH_2} \quad [109]$$

Nitrogenase reaction by NifDK (as biochemical equation not balanced for H^+):

$$\mathrm{N_2 + 8\,Fd_{red} \longrightarrow 2\,NH_3(aq) + H_2 + 8\,Fd_{ox}}, \qquad \text{requires } 8\,H^+, 16\text{ ATP} \quad [110]$$

Oxidative phosphorylation by FbcBCF and high-affine terminal oxidase FixNOPQ:

$$\mathrm{6\,QH_2 + 3\,O_2 \longrightarrow 6\,Q + 6\,H_2O} \qquad (6 \cdot 6\,H^+ = 36\,H^+ \text{ exported}) \quad [111]$$

Overall biochemical reaction, not yet balanced for charge and H^+:

$$\mathrm{2\,Arg + 2\,Mal + N_2 + 3\,O_2 + 4\,H_2O \longrightarrow 4\,Asp + 6\,NH_3 + 4\,CO_2 + H_2} \qquad \text{biochem. eq.} \quad [112]$$

Balancing the reaction stoichiometry for charge and H^+:

$$\mathrm{2\,C_6H_{15}N_4O_2{}^+ + 2\,C_4H_4O_5{}^{2-} + N_2 + 3\,O_2 + 4\,H_2O \longrightarrow 4\,C_4H_6NO_4{}^- + 6\,NH_4{}^+ + 4\,CO_2 + H_2} \qquad \text{chemical eq.} \quad [113]$$

$$\mathrm{20\,C = 20\,C} \qquad \text{C balanced} \quad [114]$$

$$\mathrm{10\,N = 10\,N} \qquad \text{N balanced} \quad [115]$$

$$\mathrm{24\,O = 24\,O} \qquad \text{O balanced} \quad [116]$$

$$\mathrm{46\,H \neq 50\,H} \qquad 4\,H^+ \text{missing} \quad [117]$$

$$-2 \neq +2 \qquad +4 \text{ missing} \quad [118]$$

Insertion of 4 H^+ to the left of 113 results in the following charge and H^+ corrected equation:

$$\mathrm{2\,C_6H_{15}N_4O_2{}^+ + 2\,C_4H_4O_5{}^{2-} + N_2 + 3\,O_2 + 4\,H_2O + 4\,H^+ \longrightarrow 4\,C_4H_6NO_4{}^- + 6\,NH_4{}^+ + 4\,CO_2 + H_2} \qquad \text{chemical eq.} \quad [119]$$

Proton translocation:

$$36\,H^+{}_{in} \longrightarrow 36\,H^+{}_{out} \qquad O_2 \text{ dependent proton translocation from 111} \quad [120]$$

$$36\,H^+{}_{out} \longrightarrow 36\,H^+{}_{in} \qquad \text{protons available from 111} \quad [121]$$

$$4\,H^+{}_{out} \longrightarrow 4\,H^+{}_{in} \qquad \text{influx of } H^+ \text{ derived from 119} \quad [122]$$

$$40\,H^+{}_{out} \longrightarrow 40\,H^+{}_{in} \qquad \text{121 + 122 \textbf{Sum of proton influx}} \quad [123]$$

Stochiometry of the proton motive force (PMF) dependent ATP synthesis:

$$\frac{[H^+]}{[ATP]} = \frac{10}{3} \quad , \frac{40}{3.33} = 12 \text{ ATP synthesized} \qquad [124]$$

Stoichiometry of Enzyme-coupled ATP synthesis:

2 ATP/ 2 Arginine generated by the action of deiminase, transcarbamoylase and carbamate kinase, 100 [125]

Sum of oxygen-dependent, oxygen-independent and Enzyme-coupled ATP synthesis - :

10.80 ATP Oxygen-dependent [126]
1.2 ATP Oxygen-independent [127]
2 ATP Enzyme-coupled [128]
14 ATP Sum [129]
-16 ATP Nitrogenase reaction [130]
-2 ATP Net loss [131]
[132]

Overall reaction of N_2 fixation by bacteroid:

$$2\,\mathrm{Arg} + 2\,\mathrm{Mal} + \mathrm{N_2} + 3\,\mathrm{O_2} + n\,\mathrm{ATP} + \text{(4-n)}\,\mathrm{H_2O} + 4\,\mathrm{H^+} \longrightarrow 4\,\mathrm{Asp} + 6\,\mathrm{NH_4}^+ + 4\,\mathrm{CO_2} + \mathrm{H_2} + \mathrm{nADP} + \mathrm{nPi} \qquad [133]$$

with n= 2 [134]

Reaction stoichiometry of N_2 fixation metabolism under the sole provision of arginine . Reaction equations are step-wise deduced from the conversion of arginine. Furthermore, reaction equations are deduced from electron bifurcation of NADH by the FixABCD complex, nitrogen fixation by nitrogenase complex and oxidative phosphorylation by the FbcBCF and the high-affine terminal oxidases FixNOPQ and CtaCDBBGE.

Model with arginine consumption through ADI pathway into glutamate and entering the TCA cycle at 2-oxoglutarate
Arginine to Ornithine:

$$\mathrm{Arg} + \mathrm{H_2O} \longrightarrow \mathrm{Cit} + \mathrm{NH_3}, \qquad [135]$$

$$\mathrm{Cit} + \mathrm{Pi} \longrightarrow \mathrm{Orn} + \mathrm{CarbP}, \qquad [136]$$

$$\mathrm{CarbP} + \mathrm{ADP} \longrightarrow \mathrm{ATP} + \mathrm{NH_3} + \mathrm{CO_2}, \qquad [137]$$

Combining 135+ 136+137

$$\mathrm{Arg} + \mathrm{H_2O} \longrightarrow \mathrm{Orn} + 2\,\mathrm{NH_3} + \mathrm{CO_2} \qquad \text{1 ATP generated from 137} \qquad [138]$$

Ornithine to Glutamate:

$$\mathrm{Orn} \longrightarrow \mathrm{Pro} + \mathrm{NH_3}, \qquad [139]$$

$$\mathrm{Pro} + \mathrm{Q} \longrightarrow \mathrm{P5C} + \mathrm{QH_2}, \qquad [140]$$

$$\mathrm{P5C} + \mathrm{H_2O} \longrightarrow \mathrm{GSA}, \qquad [141]$$

$$\mathrm{GSA} + \mathrm{NAD} + \mathrm{H_2O} \longrightarrow \mathrm{Glu} + \mathrm{NADH_2}, \qquad [142]$$

Combining 139+ 140+ 141 +142

$$\mathrm{Orn} + \mathrm{Q} + \mathrm{NAD} + 2\,\mathrm{H_2O} \longrightarrow \mathrm{Glu} + \mathrm{NH_3} + \mathrm{QH_2} + \mathrm{NADH_2} \qquad [143]$$

Arginine to Glutamate 138+ 143:

$$\mathrm{Arg} + \mathrm{Q} + \mathrm{NAD} + 3\,\mathrm{H_2O} \longrightarrow \mathrm{Glu} + 3\,\mathrm{NH_3} + \mathrm{CO_2} + \mathrm{QH_2} + \mathrm{NADH_2} \qquad \text{1 ATP generated from 137} \qquad [144]$$

Glutamate to 2-oxoglutarate:

$$\mathrm{Glu} + \mathrm{NAD} + \mathrm{H_2O} \longrightarrow \text{2-oxoglutarate} + \mathrm{NH_3} + \mathrm{NADH_2} \qquad [145]$$

Arginine to 2-oxoglutarate 144+ 145:

$$\mathrm{Arg} + \mathrm{Q} + 2\,\mathrm{NAD} + 4\,\mathrm{H_2O} \longrightarrow \text{2-oxoglutarate} + 4\,\mathrm{NH_3} + \mathrm{CO_2} + \mathrm{QH_2} + 2\,\mathrm{NADH_2} \qquad \text{1 ATP generated from 137} \qquad [146]$$

2-oxoglutarate to succinate:

$$\text{2-oxoglutarate} + \mathrm{NAD} \longrightarrow \mathrm{Succinate} + \mathrm{CO_2} + \mathrm{NADH_2} \qquad \text{1 ATP generated} \qquad [147]$$

Arginine to succinate 146+ 147:

$$\mathrm{Arg} + \mathrm{Q} + 3\,\mathrm{NAD} + 4\,\mathrm{H_2O} \longrightarrow \mathrm{Succinate} + 4\,\mathrm{NH_3} + 2\,\mathrm{CO_2} + \mathrm{QH_2} + 3\,\mathrm{NADH_2} \qquad \text{2 ATP generated from 137 and 147} \qquad [148]$$

Conversion of succinate by TCA cycle:

$$\mathrm{Succinate} + 2\,\mathrm{Q} + 5\,\mathrm{NAD} + \mathrm{ADP} + \mathrm{Pi} + 3\,\mathrm{H_2O} \longrightarrow 2\,\mathrm{QH_2} + 4\,\mathrm{CO_2} + 5\,\mathrm{NADH_2} + \mathrm{ATP} \qquad [149]$$

Metabolism of arginine through the TCA cycle 148 + 149 :

$$\mathrm{Arg} + 3\,\mathrm{Q} + 8\,\mathrm{NAD} + 10\,\mathrm{H_2O} \longrightarrow 4\,\mathrm{NH_3} + 6\,\mathrm{CO_2} + 3\,\mathrm{QH_2} + 8\,\mathrm{NADH_2} \qquad \text{3 ATP generated from 137, 147 and 149,} \qquad [150]$$

Electron bifurcation of NADH by the FixABCX complex:

$$8\,\mathrm{NADH_2} + 8\,\mathrm{Fd_{ox}} + 4\,\mathrm{Q} \longrightarrow 8\,\mathrm{NAD} + 8\,\mathrm{Fd_{red}} + 4\,\mathrm{QH_2} \qquad [151]$$

Nitrogenase reaction by NifDK (as biochemical equation not balanced for H^+):

$$\mathrm{N_2} + 8\,\mathrm{Fd_{red}} \longrightarrow 2\,\mathrm{NH_3(aq)} + \mathrm{H_2} + 8\,\mathrm{Fd_{ox}}, \qquad \text{requires } 8\,\mathrm{H^+}\text{, 16 ATP} \qquad [152]$$

Oxidative phosphorylation by FbcBCF and high affine terminal oxidase FixNOPQ and CtaCDBGE:

$$7\,\mathrm{QH_2} + 3.5\,\mathrm{O_2} \longrightarrow 7\,\mathrm{Q} + 7\,\mathrm{H_2O} \qquad (7 \cdot 6\,\mathrm{H^+} = 42\,\mathrm{H^+}\text{ exported}) \qquad [153]$$

Overall biochemical reaction (150+151+152+153), not yet balanced for charge and H^+:

$$\mathrm{Arg} + \mathrm{N_2} + 3.5\,\mathrm{O_2} + 3\,\mathrm{H_2O} \longrightarrow 6\,\mathrm{NH_3} + 6\,\mathrm{CO_2} + \mathrm{H_2} \qquad \text{biochem. eq.} \qquad [154]$$

Balancing the reaction stoichiometry for charge and H^+:

$$C_6H_{15}N_4O_2{}^+ + N_2 + 3.5\,O_2 + 3\,H_2O \longrightarrow 6\,NH_4{}^+ + 6\,CO_2 + H_2$$ chemical eq. [155]

$$6\,C = 6\,C$$ C balanced [156]

$$6\,N = 6\,N$$ N balanced [157]

$$12\,O = 12\,O$$ O balanced [158]

$$21\,H \neq 26\,H$$ 5 H^+ missing [159]

$$+1 \neq +6$$ + 5 missing [160]

Insertion of 5 H^+ to the left of 155 results in the following charge and H^+ corrected equation:

$$C_6H_{15}N_4O_2{}^+ + N_2 + 3.5\,O_2 + 3\,H_2O + 5\,H^+ \longrightarrow 6\,NH_4{}^+ + 6\,CO_2 + H_2$$ chemical eq. [161]

[162]

Proton translocation:

$$42\,H^+{}_{in} \longrightarrow 42\,H^+{}_{out}$$ O_2 dependent proton translocation from 153 [163]

$$42\,H^+{}_{out} \longrightarrow 42\,H^+{}_{in}$$ protons available from 153 [164]

$$5\,H^+{}_{out} \longrightarrow 5\,H^+{}_{in}$$ influx of H^+ derived from 161, $O_2 independent$ [165]

$$47\,H^+{}_{out} \longrightarrow 47\,H^+{}_{in}$$ 164 + 165 **Sum of proton influx** [166]

Stochiometry of the proton motive force (PMF) dependent ATP synthesis:

$$\frac{[H^+]}{[ATP]} = \frac{10}{3} \quad , \frac{47}{3.33} = 14.1$$, ATP synthesized [167]

Stoichiometry of enzyme coupled ATP synthesis:

3 ATP generated by enzyme coupled ATP synthesis of carbamoyl-pathway and TCA cycle [168]

Sum of oxygen dependent, oxygen independent and enzyme coupled ATP synthesis - :

12.6 ATP	Oxygen dependent	[169]
1.5 ATP	Oxygen independent	[170]
3 ATP	Enzyme coupled	[171]
17.2 ATP	Sum	[172]
-16 ATP	Nitrogenase reaction	[173]
**1.1 ATP**	**Net gain**	[174]
		[175]

Overall reaction of N_2 fixation by bacteroid:

$$Arg + N_2 + 3.5\,O_2 + (3\text{-}n)\,H_2O + 5\,H^+ + nADP + nPi \longrightarrow 6\,NH_4{}^+ + 6\,CO_2 + H_2 + nATP$$ [176]

with n= 1.2 [177]

Reaction stoichiometry of N_2 fixation metabolism under the assumption of an operational TCA cycle. Reaction equations are step-wise deduced from the conversion of malate and succinate, respectively. Furthermore, reaction equations are deduced from electron bifurcation of NADH by the FixABCD complex, nitrogen fixation by nitrogenase complex and oxidative phosphorylation by the FbcBCF and the high-affine terminal oxidases FixNOPQ and CtaCDBBGE.

Catabolism of malate under operational TCA cycle

$$\mathrm{Malate} + \mathrm{Q} + 5\,\mathrm{NAD} + \mathrm{ADP} + \mathrm{Pi} + 2\,\mathrm{H_2O} \longrightarrow \mathrm{QH_2} + 4\,\mathrm{CO_2} + 5\,\mathrm{NADH} + \mathrm{ATP} \quad [178]$$

multiply 178 by $\frac{8}{5}$ [179]

$$1.6\,\mathrm{Malate} + 1.6\,\mathrm{Q} + 8\,\mathrm{NAD} + 1.6\,\mathrm{ADP} + 1.6\,\mathrm{Pi} + 3.2\,\mathrm{H_2O} \longrightarrow 1.6\,\mathrm{QH_2} + 6.4\,\mathrm{CO_2} + 8\,\mathrm{NADH} + 1.6\,\mathrm{ATP} \quad [180]$$

Electron bifurcation of NADH by the FixABCX complex:

$$8\,\mathrm{NADH_2} + 8\,\mathrm{Fd_{ox}} + 4\,\mathrm{Q} \longrightarrow 8\,\mathrm{NAD} + 8\,\mathrm{Fd_{red}} + 4\,\mathrm{QH_2} \quad [181]$$

Nitrogenase reaction by NifDK (as biochemical equation not balanced for H^+):

$$\mathrm{N_2} + 8\,\mathrm{Fd_{red}} \longrightarrow 2\,\mathrm{NH_3(aq)} + \mathrm{H_2} + 8\,\mathrm{Fd_{ox}}, \qquad \text{requires } 8\,\mathrm{H^+},\ 16\ \mathrm{ATP} \quad [182]$$

Oxidative phosphorylation by FbcBCF and high-affine terminal oxidase FixNOPQ:

$1.6\,\mathrm{QH_2} + 4\,\mathrm{QH_2} = 5.6\,\mathrm{QH_2}$,Sum of QH_2 production by TCA cycle 180 and electron bifurcation 181 [183]

$$5.6\,\mathrm{QH_2} + 2.8\,\mathrm{O_2} \longrightarrow 5.6\,\mathrm{Q} + 5.6\,\mathrm{H_2O} \qquad (5.6 \cdot 6\,\mathrm{H^+} = 33.6\,\mathrm{H^+}\ \text{exported}) \quad [184]$$

Overall biochemical reaction, not yet balanced for charge and H^+:

$$1.6\,\mathrm{malate} + \mathrm{N_2} + 2.8\,\mathrm{O_2} \longrightarrow 2\,\mathrm{NH_3} + 6.4\,\mathrm{CO_2} + \mathrm{H_2} + 0.8\,\mathrm{H_2O} \qquad \text{biochem. eq.} \quad [185]$$

Balancing the reaction stoichiometry for charge and H^+:

$$1.6\,\mathrm{C_4H_4O_5}^{2-} + \mathrm{N_2} + 2.8\,\mathrm{O_2} \longrightarrow 2\,\mathrm{NH_4}^{+} + 6.4\,\mathrm{CO_2} + \mathrm{H_2} + 0.8\,\mathrm{H_2O} \qquad \text{chemical eq.} \quad [186]$$

$$6.4\,\mathrm{C} = 6.4\,\mathrm{C} \qquad \text{C balanced} \quad [187]$$

$$2\,\mathrm{N} = 2\,\mathrm{N} \qquad \text{N balanced} \quad [188]$$

$$13.6\,\mathrm{O} = 13.6\,\mathrm{O} \qquad \text{O balanced} \quad [189]$$

$$6.4\,\mathrm{H} \neq 11.6\,\mathrm{H} \qquad \text{5.2 Hmissing} \quad [190]$$

$$-3.2 \neq +2 \qquad \text{+ 5.2 missing} \quad [191]$$

Insertion of 5.2 H^+ to the left of 186 results in the following charge and H^+ corrected equation:

$$1.6\,\mathrm{C_4H_4O_5}^{2-} + \mathrm{N_2} + 2.8\,\mathrm{O_2} + 5.2\,\mathrm{H^+} \longrightarrow 2\,\mathrm{NH_4}^{+} + 6.4\,\mathrm{CO_2} + \mathrm{H_2} + 0.8\,\mathrm{H_2O}\text{chemical eq.} \quad [192]$$

Proton translocation:

$$33.6\,\mathrm{H^+}_{\mathrm{in}} \longrightarrow 33.6\,\mathrm{H^+}_{\mathrm{out}} \qquad \text{O}_2 \text{ dependent proton translocation from 184} \quad [193]$$

$$33.6\,\mathrm{H^+}_{\mathrm{out}} \longrightarrow 33.6\,\mathrm{H^+}_{\mathrm{in}} \qquad \text{protons available from 184} \quad [194]$$

$$5.2\,\mathrm{H^+}_{\mathrm{out}} \longrightarrow 5.2\,\mathrm{H^+}_{\mathrm{in}} \qquad \text{influx of H}^+ \text{ derived from 192} \quad [195]$$

$$38.8\,\mathrm{H^+}_{\mathrm{out}} \longrightarrow 38.8\,\mathrm{H^+}_{\mathrm{in}} \qquad \text{194 + 195 \textbf{Sum of proton influx}} \quad [196]$$

Stochiometry of the proton motive force (PMF) dependent ATP synthesis:

$$\frac{[\mathrm{H^+}]}{[\mathrm{ATP}]} = \frac{10}{3} \qquad , \frac{38.8}{3.33} = 11.64 \text{ , ATP synthesized} \quad [197]$$

Stoichiometry of Enzyme-coupled ATP synthesis:

1.6 ATP/ malate generated by succinyl-CoA synthase, 178 [198]

Sum of oxygen-dependent, oxygen-independent and Enzyme-coupled ATP synthesis - :

10.08 ATP Oxygen-dependent [199]

1.56 ATP Oxygen-independent [200]

1.6 ATP Enzyme-coupled [201]

13.42 ATP Sum [202]

-16 ATP Nitrogenase reaction [203]

-2.76 ATP Net loss [204]

[205]

Overall reaction of N_2 fixation by bacteroid:

$$1.6\,\mathrm{Malate} + \mathrm{N_2} + 2.8\,\mathrm{O_2} + 5.2\,\mathrm{H^+} + 2.76\,\mathrm{ATP} + 1.96\,\mathrm{H_2O} \longrightarrow 2\,\mathrm{NH_4}^{+} + 6.4\,\mathrm{CO_2} + \mathrm{H_2}\,2.36\,\mathrm{ADP} + 2.36\,\mathrm{Pi} \quad [206]$$

With malate as the sole energy source, a net loss of -2.76 ATP occurs, therefore malate is likely not acting as the sole substrate of symbiotic nitrogen fixation.

Model B: Succinate and operational TCA cycle
Conversion of succinate by TCA cycle:

$$\mathrm{Succinate} + 2\,\mathrm{Q} + 5\,\mathrm{NAD} + \mathrm{ADP} + \mathrm{Pi} + 3\,\mathrm{H_2O} \longrightarrow 2\,\mathrm{QH_2} + 4\,\mathrm{CO_2} + 5\,\mathrm{NADH} + \mathrm{ATP} \quad [207]$$

$$\text{multiply } 207 \text{ by } \frac{8}{5} \quad [208]$$

$$1.6\,\mathrm{Succinate} + 3.2\,\mathrm{Q} + 8\,\mathrm{NAD} + 1.6\,\mathrm{ADP} + 1.6\,\mathrm{Pi} + 4.8\,\mathrm{H_2O} \longrightarrow 3.2\,\mathrm{QH_2} + 6.4\,\mathrm{CO_2} + 8\,\mathrm{NADH} + 1.6\,\mathrm{ATP} \quad [209]$$

Electron bifurcation of NADH by the FixABCX complex:

$$8\,\mathrm{NADH_2} + 8\,\mathrm{Fd_{ox}} + 4\,\mathrm{Q} \longrightarrow 8\,\mathrm{NAD} + 8\,\mathrm{Fd_{red}} + 4\,\mathrm{QH_2} \quad [210]$$

Nitrogenase reaction by NifDK (as biochemical equation not balanced for H^+):

$$\mathrm{N_2} + 8\,\mathrm{Fd_{red}} \longrightarrow 2\,\mathrm{NH_3(aq)} + \mathrm{H_2} + 8\,\mathrm{Fd_{ox}}, \qquad \text{requires } 8\,\mathrm{H^+}\text{, 16 ATP} \quad [211]$$

Oxidative phosphorylation by FbcBCF and high-affine terminal oxidase FixNOPQ:

$$3.2\,\mathrm{QH_2} + 4\,\mathrm{QH_2} = 7.2\,\mathrm{QH_2}, \text{Sum of } \mathrm{QH_2} \text{ production by TCA cycle } 209 \text{ and electron bifurcation } 210 \quad [212]$$

$$7.2\,\mathrm{QH_2} + 3.6\,\mathrm{O_2} \longrightarrow 7.2\,\mathrm{Q} + 7.2\,\mathrm{H_2O} (7.2 \cdot 6\,\mathrm{H^+} = 43.2\,\mathrm{H^+} \text{ exported}) \quad [213]$$

Overall biochemical reaction, not yet balanced for charge and H^+:

$$1.6\,\mathrm{succinate} + \mathrm{N_2} + 3.6\,\mathrm{O_2} \longrightarrow 2\,\mathrm{NH_3} + 6.4\,\mathrm{CO_2} + \mathrm{H_2} + 0.8\,\mathrm{H_2O} \qquad \text{biochem. eq.} \quad [214]$$

Balancing the reaction stoichiometry for charge and H^+:

$$1.6\,\mathrm{C_4H_4O_4}^{2-} + \mathrm{N_2} + 3.6\,\mathrm{O_2} \longrightarrow 2\,\mathrm{NH_4}^{+} + 6.4\,\mathrm{CO_2} + \mathrm{H_2} + 0.8\,\mathrm{H_2O} \qquad \text{chemical eq.} \quad [215]$$

$$6.4\,\mathrm{C} = 6.4\,\mathrm{C} \qquad \text{C balanced} \quad [216]$$

$$2\,\mathrm{N} = 2\,\mathrm{N} \qquad \text{N balanced} \quad [217]$$

$$13.6\,\mathrm{O} = 13.6\,\mathrm{O} \qquad \text{O balanced} \quad [218]$$

$$6.4\,\mathrm{H} \neq 11.6\,\mathrm{H} \qquad 5.2\,\mathrm{H^+} \text{ missing} \quad [219]$$

$$-3.2 \neq +2 \qquad +\,5.2 \text{ missing} \quad [220]$$

Insertion of 5.2 H^+ to the left of 215 results in the following charge and H^+ corrected equation:

$$1.6\,\mathrm{C_4H_4O_4}{-}2 + \mathrm{N_2} + 2.8\,\mathrm{O_2} + 5.2\,\mathrm{H^+} \longrightarrow 2\,\mathrm{NH_4}^{+} + 6.4\,\mathrm{CO_2} + \mathrm{H_2} + 0.8\,\mathrm{H_2O} \qquad \text{chemical eq.} \quad [221]$$

Proton translocation:

$$43.2\,\mathrm{H^+}_{\mathrm{in}} \longrightarrow 43.2\,\mathrm{H^+}_{\mathrm{out}} \qquad \mathrm{O_2} \text{ dependent proton translocation from } 184 \quad [222]$$

$$43.2\,\mathrm{H^+}_{\mathrm{out}} \longrightarrow 43.2\,\mathrm{H^+}_{\mathrm{in}} \qquad \text{protons available from } 184 \quad [223]$$

$$5.2\,\mathrm{H^+}_{\mathrm{out}} \longrightarrow 5.2\,\mathrm{H^+}_{\mathrm{in}} \qquad \text{influx of } \mathrm{H^+} \text{ derived from } 221 \quad [224]$$

$$48.8\,\mathrm{H^+}_{\mathrm{out}} \longrightarrow 48.8\,\mathrm{H^+}_{\mathrm{in}} \qquad 223 + 224 \ \textbf{Sum of proton influx} \quad [225]$$

Stochiometry of the proton motive force (PMF) dependent ATP synthesis:

$$\frac{[\mathrm{H^+}]}{[\mathrm{ATP}]} = \frac{10}{3}, \quad \frac{48.8}{3.33} = 14.64, \text{ ATP synthesized} \quad [226]$$

Stoichiometry of Enzyme-coupled ATP synthesis:

1.6 ATP generated by Enzyme-coupled ATP synthesis of TCA cycle [227]

Sum of oxygen-dependent, oxygen-independent and Enzyme-coupled ATP synthesis - :

12.96 ATP Oxygen-dependent [228]
1.56 ATP Oxygen-independent [229]
1.6 ATP Enzyme-coupled [230]
16.12 ATP Sum [231]
-16 ATP Nitrogenase reaction [232]
0.12 ATP Net gain [233]

Overall reaction of N_2 fixation by bacteroid:

$$1.6\,\mathrm{succinate} + \mathrm{N_2} + 3.6\,\mathrm{O_2} + 5.2\,\mathrm{H^+} + 0.12\,\mathrm{ADP} + 0.12\,\mathrm{Pi} \longrightarrow 2\,\mathrm{NH_4}^{+} + 6.4\,\mathrm{CO_2} + \mathrm{H_2} + 0.12\,\mathrm{ATP} + 0.92\,\mathrm{H_2O} \quad [234]$$

With Succinate as the sole energy source, net production of 0.12 ATP occurs. However, net ATP gain does not occur if more than 0.4 protons out of a total of 48.8 per N_2 fixed are transported by membrane diffusion rather than by the action of ATP synthase. Over 99.1% of the protons generated must pass the ATP synthase complex, otherwise, no ATP gain is achieved. Such efficiency is likely not realistic for a biochemical process giving the high permeability of the biological cell membrane for protons.
