# Supplementary material for: Co‐catabolism of arginine and succinate drives symbiotic nitrogen fixation
Source: Mol Syst Biol. 2020 Jun 3;16(6):e9419. doi: 10.15252/msb.20199419 (PMC7268258; doi:10.15252/msb.20199419)
Supplement: Supplementary file 2 — Expanded View Figures PDF [file MSB-16-e9419-s002.pdf]

# Expanded View Figures

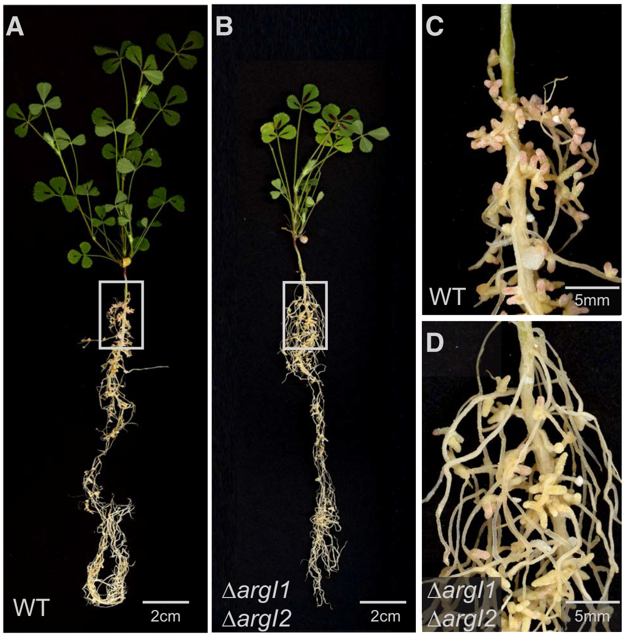


**Figure EV1. Symbiotic defects of *S. meliloti* mutants impaired in arginine catabolism.**

A Phenotype of *M. truncatula lss* plants inoculated with *S. meliloti* WT.

B Phenotype of *M. truncatula lss* plants inoculated with *S. meliloti* double deletion mutant Δ*argI1*, Δ*argI2*.

C Root-nodule phenotype of *M. truncatula lss* plants inoculated with *S. meliloti* WT.

D Root-nodule phenotype of *M. truncatula lss* plants inoculated with *S. meliloti* double deletion mutant Δ*argI1*, Δ*argI2*.

© 2020 The Authors
